# STM study on the self-assembly of oligothiophene-based organic semiconductors

**DOI:** 10.3762/bjnano.2.88

**Published:** 2011-12-07

**Authors:** Elena Mena-Osteritz, Marta Urdanpilleta, Erwaa El-Hosseiny, Berndt Koslowski, Paul Ziemann, Peter Bäuerle

**Affiliations:** 1 Institute of Organic Chemistry II and Advanced Materials, Ulm University, Albert-Einstein-Allee 11, D 89081 Ulm, Germany; 2 Department of Applied Physics, University of the Basque Country (UPV/EHU), Plaza de Europa, 1, 20018 Donostia - San Sebastián, Spain; 3 Department of Solid State Physics, Ulm University, Albert-Einstein-Allee 11, D 89081 Ulm, Germany

**Keywords:** 2-D crystals, functionalized oligothiophenes, H-bonding, intermolecular interaction, scanning tunneling microscopy

## Abstract

The self-assembly properties of a series of functionalized regioregular oligo(3-alkylthiophenes) were investigated by using scanning tunneling microscopy (STM) at the liquid–solid interface under ambient conditions. The characteristics of the 2-D crystals formed on the (0001) plane of highly ordered pyrolitic graphite (HOPG) strongly depend on the length of the π-conjugated oligomer backbone, on the functional groups attached to it, and on the alkyl substitution pattern on the individual thiophene units. Theoretical calculations were performed to analyze the geometry and electronic density of the molecular orbitals as well as to analyze the intermolecular interactions, in order to obtain models of the 2-D molecular ordering on the substrate.

## Introduction

In the last few decades conjugated organic polymers and oligomers have attracted a broad interest due to their excellent electronic and transport properties in the solid state, which allow their application in a variety of organic-electronic devices [1]. Among others, organic semiconductors based on thiophenes are very promising materials in the field, because of their superior stability and the possibility to chemically functionalize them without affecting their electronic properties. The most prominent example is given by the regioregular (head-to-tail-coupled) poly(3-hexylthiophene), which is among the best-performing photoactive materials in polymer solar cells [2–4]. The self-organization of the polymer chains in the bulk seems to be a key factor, which determines the efficiency of the charge transport through the material and ultimately the performance in organic-electronic devices. In recent years, various approaches have been followed to elucidate the ordering principles of conjugated polymers, by X-ray diffraction and scanning tunneling microscopy. Valuable information about intermolecular interactions taking place on substrates has been obtained [5–7].

Although the substitution pattern is regioregular and chemically controlled, the inherent chain length dispersity of poly(3-alkylthiophenes) leads to a mesoscopic structure of the resulting films, which are composed of polycrystalline domains embedded in a disordered matrix [7]. Furthermore, the knowledge of clear structure–property relationships, connecting the physical properties with the length of the conjugated system, and the recent success in the field of small-molecule organic devices have restored huge interest in structurally well-defined and therefore monodisperse, crystalline oligomers [8].

The method of choice to investigate the ordering of organic semiconductors adsorbed on flat metallic surfaces, at the desired molecular level in the subnanometer range, is scanning tunneling microscopy (STM). With this method, the self-assembly of oligo- and polythiophenes on surfaces has been successfully investigated in the last few years [5–6,9–14]. The 2-D crystalline arrangement on surfaces is a result of a delicate balance between several weak intermolecular van der Waals forces and molecule–substrate interactions, as well as intermolecular hydrogen bonding in the case of functionalized oligothiophenes [15–17]. The typical flat metallic substrates (HOPG, Au(111), Ag(111), etc.) employed in STM differ from the ITO electrodes used in the devices, which are usually rendered flat by an organic hole-transporting layer. Despite the differences, a good approach to elucidate the bulk properties of the active molecules is to probe their intermolecular interactions on nonreactive substrates, such as HOPG, by means of STM.

In this paper, we report the investigation of the self-assembly properties of a series of carbonic acid functionalized regioregular (head-to-tail-coupled) oligo(3-hexylthiophenes) on HOPG at the liquid–solid interface as examined by STM at room temperature. The ambient and environmental conditions employed in this study are essential in order to mimic the deposition conditions for real devices. Supported by quantum-chemical calculations, the interpretation of the STM images and the analysis of the intermolecular forces will be highlighted.

## Results and Discussion

**Figure 1:**
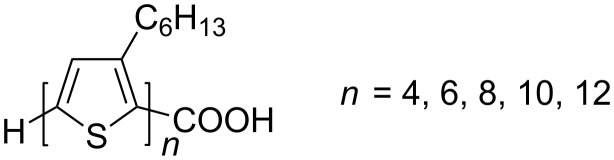
General formula of carboxylic acid functionalized oligothiophenes **H*n*TCOOH**.

The molecules analyzed in this study, carboxylic acid-functionalized regioregular oligo(3-hexylthiophenes), **H*n*TCOOH**, are sketched in Figure 1. The π-conjugated system, comprising the aromatic thiophene rings and being responsible for the electronic properties of the molecule, extends from 4 to 12 thiophene repeating units, and the molecular size stretches accordingly from 1.7 nm to 5.4 nm. The carboxylic acid group (**COOH**) at the terminal α-position of the oligothiophene backbone allows the formation of effective intermolecular hydrogen bonding through dimer formation. The regioregular hexyl-side-chain substitution pattern enhances the solubility of the compounds in organic solvents and their crystallinity on the substrate.

**Figure 2:**
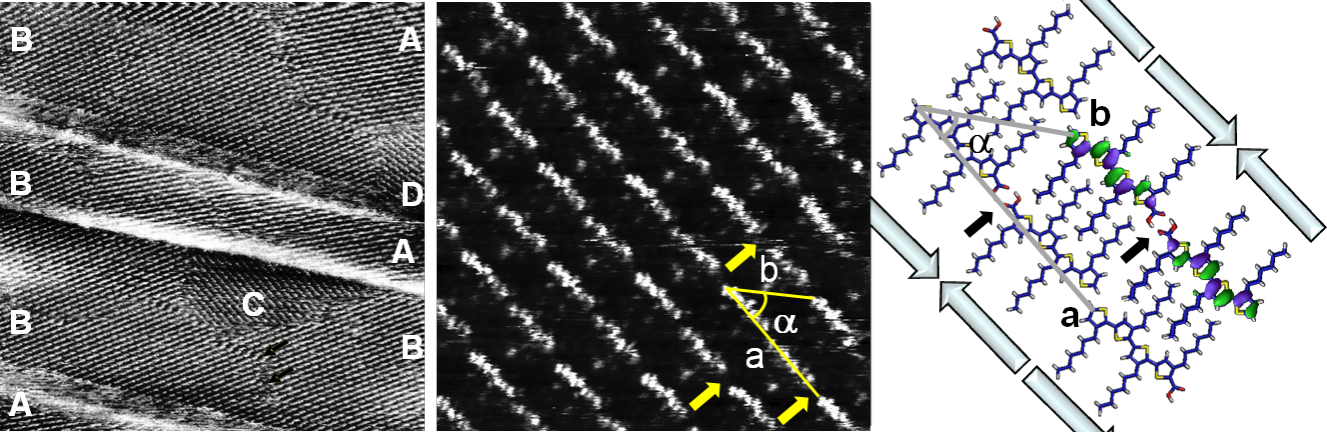
Left: STM image of **H4TCOOH** on HOPG (100 × 100 nm^2^, *U* = −120 mV, *I* = 50 pA). The letters label the different domains as explained in the text. Center: STM current image of **H4TCOOH** on HOPG (10 × 10 nm^2^, *U* = −100 mV, *I* = 50 pA). The arrows label the positions of the carboxylic groups. Right: Quantum chemical model of the adsorbate, including the electron-density distribution of the HOMO and HOMO−1 for the dimer.

Figure 2 shows a large-scale STM image of a monolayer of tetramer **H4TCOOH** adsorbed at the solution–HOPG interface. Several terraces of the graphite substrate are visible in the image. On the terraces, a very well ordered monolayer of **H4TCOOH** can be seen extending over the micrometer range.

In the first images after deposition, several domains with sizes ranging from tens to hundreds of nanometers can be recognized. In Figure 2 (left) four out of six possible domains (labeled “A” to “D”) can be seen. The six domains correspond to the two enantiomorphic molecular arrangements (with a relative angle of 10°) combined with the three crystallographic axes of the underlying HOPG substrate. In Figure 2 (left) “A” and “C” correspond to two orientations (at an angle of 120°) of the same enantiomorph, as do “B” and “D”.

The molecules are lying flat on the nonreactive HOPG surface. The oligothiophene backbone carrying the delocalized π-electron system is recognized in STM images as bright spots under the given scanning conditions, which correspond to a higher tunneling probability. On the contrary, the alkyl side chains, corresponding to the insulating part of the molecules, are extended perpendicular to the oligothiophene backbone and can be recognized as dark regions, reflecting the expected lower tunneling probability.

Figure 2 (center) shows a short range image of **H4TCOOH** adsorbed on HOPG, exhibiting submolecular resolution. The unit cell contains two molecules and the cell parameters are *a* = 3.6 ± 0.1 nm, *b* = 2.1 ± 0.1 nm and α = 42 ± 2° (Table 1). The molecules are ordered in lamellae with an interlamellar distance of 1.4 nm. This value correlates very well with data published for ordered monolayers of hexyl-substituted oligo- and polythiophenes and can be explained by van der Waals interaction driven full interdigitation of the alkyl chains of two opposite molecules in their all-*trans* conformation in different rows [9–12,18].

**Table 1:** STM and theoretical parameters of 2-D crystals of the investigated carboxylic acid functionalized oligothiophenes **H*n*TCOOH**.

Compound	Molecular and Lattice Parameters	STM	Calculations

**H4TCOOH**	Molecule length [nm] *a* [nm] *b* [nm] α [°]	1.7 3.6 2.1 42	1.8 3.6 2.1 39

**H6TCOOH**	Molecule length [nm] *a* [nm] *b* [nm] α [°]	2.6 5.3 3.0 29	2.6 5.4 2.9 27

**H8TCOOH**	Molecule length [nm] *a* [nm] *b* [nm] α [°]	3.5 7.0 3.8 21	3.4 6.8 3.7 22

**H10TCOOH**	Molecule length [nm] *a* [nm] *b* [nm] α [°]	4.3 9.0 4.2 19	4.2 8.5 4.5 18

**H12TCOOH**	Molecule length [nm] *a* [nm] *b* [nm] α [°]	4.6 9.6 4.9 17	4.7 9.5 4.9 16

Theoretical calculations on a group of four molecules, excluding the substrate, support the dimer formation and are in very good agreement with the observed unit-cell parameters, with *a* = 3.6 ± 0.1 nm, *b* = 2.1 ± 0.1 nm and α = 39 ± 1° (Figure 2, right, and Table 1). In the lamellae the molecules form an angle of 8° between the molecular axis and the row direction. The two molecules building the pair are separated by small regions of low tunneling current (labeled with an arrow in Figure 2, center), which we assign to the carboxylic acid ends of two **H4TCOOH** molecules in a head-to-head arrangment. It is widely observed in STM images that carboxylic acid groups appear as dark spots under negative bias [19–20]. Our theoretical calculations support the experimental findings (Figure 2, right), showing no contribution of the end-function to the occupied frontier orbitals (HOMO and HOMO−1) close to the Fermi level. This arrangement stabilizes the monolayer by neutralizing the dipole moments of the molecules, which are oriented along the molecular axis (Figure 2, right; plain arrows). The carboxylic acid groups of two head-to-head arranged molecules are able to undergo hydrogen-bond formation, additionally stabilizing the monolayer (Figure 2, right).

In the current image (Figure 2, center) submolecular resolution was obtained for the oligothiophene backbones. Due to the low negative bias applied and at the limit given by the tunneling barrier of this compound, we can assume that the observed eight lobes per molecule correspond to the local density of states (LDOS) of the molecule, which are very close to the Fermi level and are coupled, at this bias, to an almost anisotropic contribution from the LDOS of the substrate.

**Figure 3:**
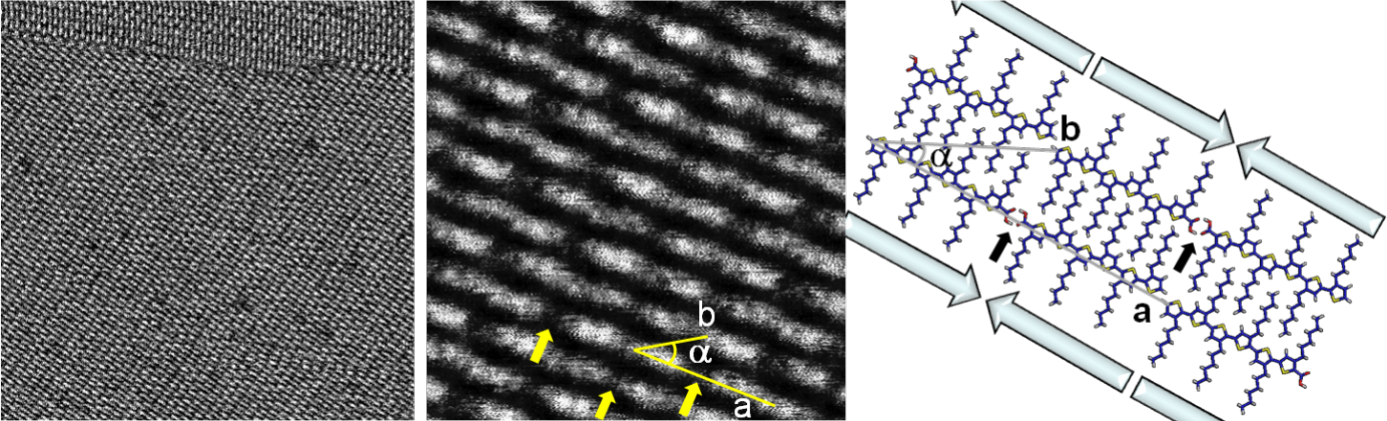
STM image of **H6TCOOH**. Left: 70 × 70 nm^2^, *U* = −360 mV, *I* = 50 pA. Center: 20 × 20 nm^2^, *U* = −361 mV, *I* = 50 pA. Right: Theoretical model. The small black arrows emphasize the H-bonding position. The big arrows indicate the orientation of the molecular dipole moments.

In Figure 3, STM images of a 2-D crystal of the next higher homologue, hexamer **H6TCOOH**, are depicted. Figure 3, left, shows a large-scale STM image of a densely packed monolayer. The molecules are perfectly arranged in lamellae and two domains (out of six possibilities, see above) can be observed. The lamellae are separated by about 1.3 nm, like in the case of **H4TCOOH.** In these regions the insulating hexyl chains interdigitate (Figure 3, right). Despite a high degree of crystallinity, the monolayer reveals a few defects (Figure 3, left), which are noticeable as faint depressions, probably related to molecules adsorbed at unstable sites. Adsorption and desorption processes equilibrating at the liquid–solid interface induce, in turn after 10–20 minutes, a self-healing process to form a perfectly ordered monolayer over several micrometers.

Figure 3 (center) shows a small-scale image of a single domain. The molecular resolution allows the determination of the unit-cell parameters: *a* = 5.3 ± 0.1 nm, *b* = 3.0 ± 0.1 nm and α = 29 ± 2°. In the unit cell two molecules arrange as a dimer, with their carboxylic acid functional groups facing each other due to H-bonding, like in the case of **H4TCOOH**. With the help of theoretical calculations a model of the molecular packing can be obtained (Figure 3, right). The unit-cell parameters are calculated to be *a* = 5.4 nm, *b* = 2.9 nm, and α = 27° in very good agreement with the experimental data. The plain arrows depicted in the model show that this molecular arrangement allows the full neutralization of the molecular dipoles within the plane.

**Figure 4:**
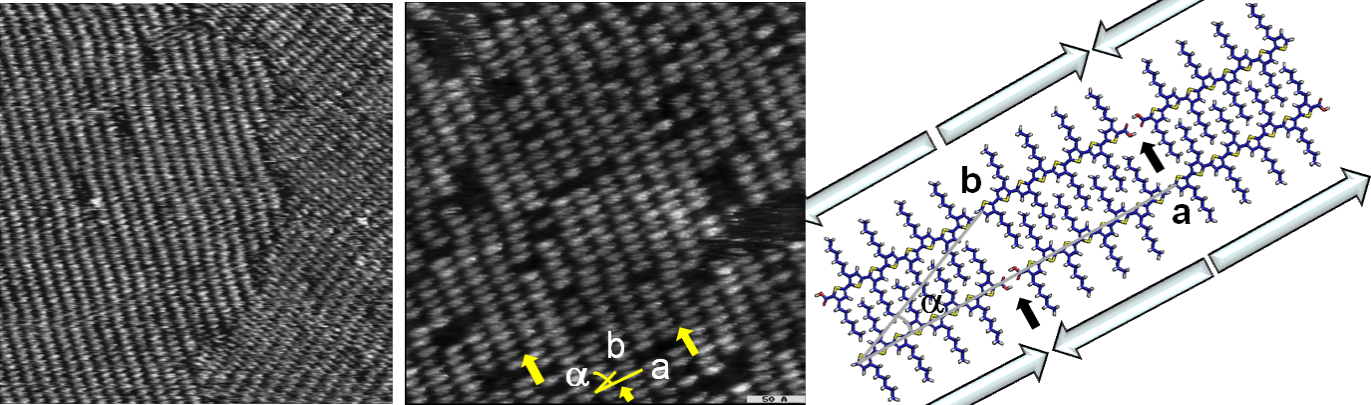
STM images of **H8TCOOH**. Left: 60 × 60 nm^2^, *U* = −640 mV, *I* = 44 pA. Center: 30 × 30 nm^2^, *U* = −725 mV, *I* = 35 pA. Right: Theoretical model. The small black arrows indicate the H-bonds. The big arrows show the orientation of the molecular dipole moments.

**Figure 5:**
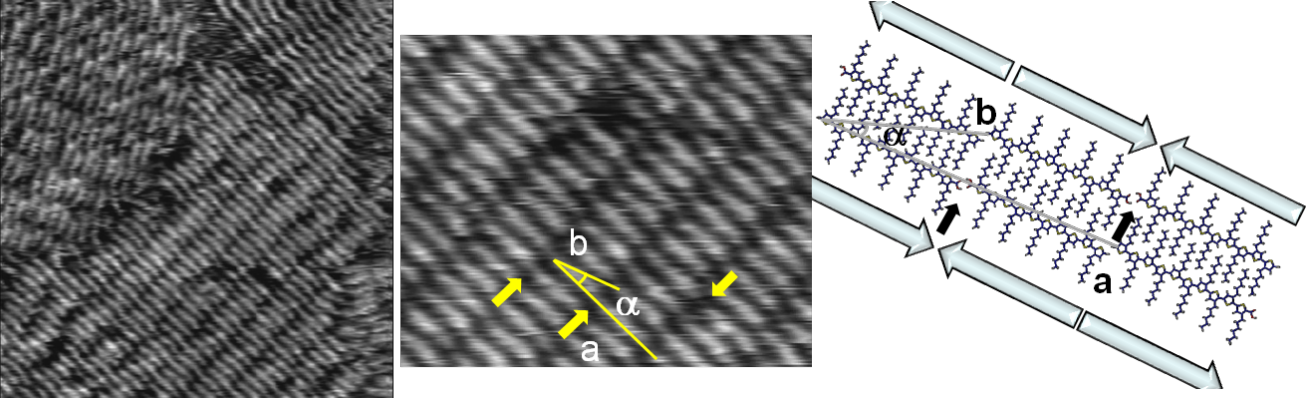
STM images of **H10TCOOH**. Left: 50 × 50 nm^2^, *U* = −200 mV, *I* = 73 pA. Center: 24 × 19 nm^2^, *U* = −100 mV, *I* = 22 pA. Right: Theoretical model. The small black arrows point out the H-bonding position. The big arrows show the orientation of the molecular dipole moments.

In Figure 4 and Figure 5, STM images of compounds **H8TCOOH** and **H10TCOOH** adsorbed at the HOPG surface are shown. The large-scale images (Figure 4 and Figure 5, left) reveal monolayers with different orientations (see above). The STM image of octamer **H8TCOOH** was taken immediately after adsorption and therefore reveals an increased number of vacancies relative to monolayers of the shorter oligomers. This phenomenon can be explained by a decrease in diffusivity as the size of the oligomers increase. However, the adsorption–desorption dynamics at the surface and the observed Ostwald ripening [21–22] lead to an almost perfect monolayer with time. The arrangement of the molecules reveals a more columnar than lamellar ordering, although after careful analysis, the dimer formation can also be determined for these long oligomers (Figure 4 and Figure 5, center). The unit cell calculated for the crystalline regions of **H8TCOOH** and **H10TCOOH** monolayers contains two molecules and the parameters correlate very well with the quantum-chemical model displayed in Figure 4 and Figure 5, right, and in Table 1. The more extended π-system of **H8TCOOH** and **H10TCOOH** induces a face-to-face molecular arrangement with a negligible offset and therefore a more columnar structure. The distance between the columns amounts to 1.3 nm and the space is filled by interdigitating alkyl chains (Figure 4 and Figure 5, right).

**Figure 6:**
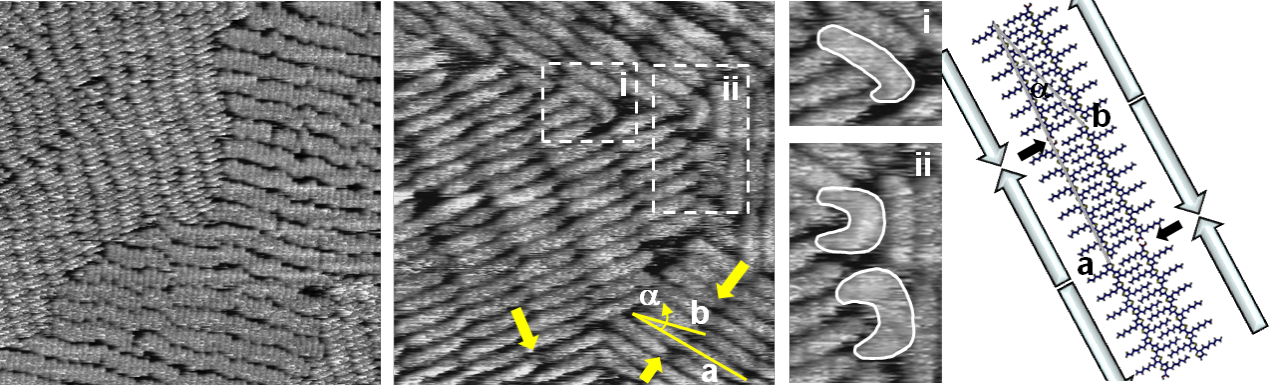
STM images of **H12TCOOH**. Left: 80 × 80 nm^2^, *U* = −200 mV, *I* = 73 pA. Center: 20 × 20 nm^2^, *U* = −750 mV, *I* = 68 pA. Details of the regions in the dashed rectangles are given in (i) and (ii). Right: Theoretical model. The small black arrows point out the H-bonding position. The big arrows show the orientation of the molecular dipole moments.

The 2-D crystal of the longest oligomer, **H12TCOOH**, is shown in Figure 6, left. A densely packed monolayer can be seen. One can easily recognize the molecules, which are arranged in columns and domains related to the crystallographic axes of the HOPG substrate. The columns are not perfectly straight, in contrast to the stacks of derivatives **H8TCOOH** and **H10TCOOH**, due to several possibilities for an offset in the pseudo face-to-face arrangement of the long **H12TCOOH** molecules.

In the small-scale STM images of **H12TCOOH** some very interesting effects can be seen (Figure 6, center). At the domain boundaries, molecules in a nonlinear, bent shape can be distinguished (Figure 6, center right (i) and (ii)). In order to fill the space between the domains, the adsorbed molecules partially change their conformation from the usual linear shape to a crescent shape. In Figure 6, center right (i), the five terminal thiophenes are arranged in a *syn*-conformation causing the observed hairpin bend. In Figure 6, center right (ii), an oligomer conformation, in which several thiophene units are in *syn-*arrangement, seems to be responsible for the observed crescent shape of the adsorbed **H12TCOOH** molecules. The hairpin conformation was already observed for corresponding regioregular poly(3-alkylthiophenes) adsorbed on a HOPG surface [5], but for an oligothiophene this behavior is shown here for the first time. More detailed analysis on the adsorption conformation at different solution concentrations of all the oligomers presented in this communication reveals that only the longest oligothiophene, **H12TCOOH**, changes the typical all-*anti* conformation to *syn*-containing ones at the domain boundaries. X-ray structure analyses on different series of alkylated oligothiophenes [12,18] have shown that, with a unique exception, smaller oligomers throughout prefer an all-*anti* conformation [23], whereas for longer oligomers *syn-*conformations at the terminal thiophene units typically seem to be more favored.

## Conclusion

The self-assembling properties of a series of regioregularly alkylated oligothiophenes on HOPG were studied by STM at the solid–liquid interface. The experimental unit-cell parameters were compared with the results of quantum chemical calculations and a very good agreement was found. This result demonstrates a major contribution of intermolecular van der Waals and H-bonding interactions to the stabilization of the monolayer on the HOPG surface. Conformational changes of the **H12TCOOH** molecules at the domain boundaries of the adsorbate were shown for the first time for self-assembling oligothiophenes. This effect appears to be closely related to the already known hairpin conformation of the related regioregular poly(3-alkylthiophenes).

## Experimental

The solutions were freshly prepared with 1,2,4-trichlorobenzene (Aldrich). STM measurements were carried out under ambient conditions with a low-current STM (RHK Rochester Hills, Michigan, USA) equipped with an STM 1000 control system. Details concerning the set-point current and bias applied are given in the figure captions. Mechanically cut Pt/Ir (80/20) tips were used. For the measurements at the solution–substrate interface, a solution of the compound in 1,2,4-trichlorobenzene was applied onto a freshly cleaved (0001) face of highly oriented pyrolytic graphite (HOPG). Measurements were repeated with different tips and different samples to confirm reproducibility and to ensure that the images were not affected by tip and sample artifacts. Theoretical calculations were performed on a semi-empirical basis with the Austin Model 1 (AM1) and the Parameterized Model 3 (PM3) by using the Hyperchem (Hypercube, Inc., FL) software package. No constraints or annealing simulations were applied.
